# Time and quality in and out of class: the roles of instruction and homework in mathematics achievement and interest

**DOI:** 10.3389/fpsyg.2026.1870861

**Published:** 2026-06-23

**Authors:** Kexin Qin, Yehui Wang

**Affiliations:** Collaborative Innovation Center of Assessment for Basic Education Quality, Beijing Normal University, Beijing, China

**Keywords:** homework quality, homework time, instructional clarity, instructional time, mathematics achievement, mathematics interest

## Abstract

**Introduction:**

How to support students' mathematics achievement while also maintaining their mathematics interest has become a central concern. Grounded in Carroll's model of school learning, the present study focuses on two central learning activities of classroom instruction and out-of-class homework to investigate how the factors of time and quality within these activities are associated with students' mathematics achievement and interest.

**Methods:**

Participants were 2808 eighth-grade students in China (45.5% female) from 76 schools across the nine counties. Students completed a questionnaire that included background information and scales measuring mathematics interest, instructional time, instructional clarity, homework time, and homework quality, as well as a mathematics achievement test. A series of multilevel regression models were conducted to examine the effects of instructional time, instructional clarity, homework time, and homework quality on mathematics achievement and mathematics interest.

**Results:**

The study revealed that at the within-school level, mathematics achievement was positively associated with instructional clarity and non-linearly related to instructional time. Higher homework quality reduced the achievement gains of spending 60–90 min on homework compared with spending less than 30 min. Between-school effects were either non-significant or lacked substantial practical meaning. For mathematics interest, instructional clarity and homework quality played a more prominent role than instructional time and homework time. Instructional clarity strengthened the positive association between instructional time and mathematics interest. Moreover, mathematics interest was positively related to homework quality while negatively related to homework time.

**Discussion:**

These findings provide insights into how time and quality in instructional and homework contexts are related to students' mathematics achievement and interest. They suggested that the quality-related factors (instructional clarity and homework quality) are more practically relevant to students' development of mathematics achievement and mathematics interest.

## 1 Introduction

Mathematics is one of the basic subjects in compulsory education. Learning mathematics during middle school plays a critical role in preparing students to acquire advanced knowledge and has a profound influence on their future success in science and technology fields (Claessens and Engel, 2013; OECD, 2023). International large-scale assessment programs consistently showed that in East Asian countries (regions), students at the lower secondary level performed well in mathematics but displayed negative attitudes toward this subject. In cycles of the Trends in International Mathematics and Science Study (TIMSS), Singapore, Chinese Taipei, Korea, Japan, and Hong Kong ranked in the top five for mathematics achievement (Mullis et al., 2004, 2020). However, Japanese and Korean students reported disliking mathematics the most (Leung, 2002), and students in Singapore, Hong Kong, and Taiwan less valued enjoying mathematics learning as an inner force (Zhu and Leung, 2011). This paradox of high achievement coexisting with low interest may be partly due to the fact that the academic excellence of East Asian students often comes at the cost of a heavy academic burden, reflected in considerably longer learning hours than the average of the Organization for Economic Co-operation and Development (OECD) (OECD, 2016; Sun, 2017).

How to support students' mathematics achievement development while also maintaining their mathematics interest has become a central concern for researchers and educators. Carroll's (1963) model of school learning provides an important perspective in this issue. According to the model, time on a given learning task is assumed to interact with the quality of the task in relation to students' learning outcomes (Carroll, 1963, 1989). The amount of time reflects students' opportunities to engage with learning, while the quality of the learning process is related to the time needed for mastery. The Carroll model has been applied to various learning contexts, including classroom instruction and out-of-class homework (Trautwein and Köller, 2003). Based on this framework, extending instructional or homework time in mathematics may be associated with higher achievement but also be related to lower mathematics interest when students experience overload. However, higher quality of instruction or homework may be associated with more efficient and less burdensome learning experiences, and correspond to favorable levels of mathematics interest (Claessens and Engel, 2013; Rivkin and Schiman, 2015).

China is one of the typical representatives of East Asian cultures, which provides a relevant context for examining this issue. In this study, we focused our analysis on students at the eighth grade for two considerations. First, the eighth grade is a critical transitional period in mathematics learning. The curriculum in this grade increases in abstraction, and research repeatedly showed students' decline in mathematics interest during this period (Frenzel et al., 2012). Second, the eighth grade is the primary target population for large-scale international assessments, and focusing on this cohort allows our findings within the Chinese context to be more comparable and generalized across global educational research.

Drawing on data from Chinese eighth-grade students, the present study focuses on two central learning activities of classroom instruction and out-of-class homework to investigate how the factors of time and quality within these activities are associated with students' mathematics achievement and interest. By doing so, the study aims to provide evidence regarding the relationship between learning time, learning quality, and students' academic and motivational outcomes in mathematics.

## 2 Literature review

### 2.1 Theoretical foundation

The model of school learning proposed by Carroll (1963) defines students' learning outcomes as the function of the time


$$\begin{array}{l} \text{Learning outcomes}=f\;\left(\frac{\text{time spent in learning}}{\text{time needed in learning}}\right)\\ =f\;\left(\frac{\left(\text{time allocated to learning}\right)\times \left(\text{perseverance}\right)}{\left(\text{aptitude}\right)\times \left(\text{ability to understand instruction}\right)\times \left(\text{the quality of instruction}\right)}\right) \end{array}$$ (1)

and quality invested in a learning task. As Equation 1 showed, learning outcomes can be represented as the ratio between “time spent in learning” and “time needed in learning,” where time spent in learning is shaped by time allocated to learning and students' perseverance, and time needed in learning is influenced by aptitude, ability to understand instruction, and the quality of instruction.

Building on this theoretical framework, researchers argued that schools can support students in meeting academic standards by allocating sufficient learning time and improving instructional quality, which helps compensate for achievement gap associated with variations in aptitude (Bloom, 1974). Importantly, Carroll (1989) highlighted time allocated to learning and quality of instruction as pivotal variables, positing that their proper management is closely related to students' perseverance.

Carroll's model conceptualized how the quality and time invested in a given task influence students' learning outcome, with most applications focusing on classroom instruction. Yet, in formal education, homework constitutes a major learning context that extends students' learning time beyond classrooms and provides additional opportunities to engage with the same content (Cooper, 2007). Trautwein and Köller (2003) expanded the Carroll model to homework research. Homework increases the time students spend on learning, whereas high-quality homework is expected to provide more structured practice and feedback, which may be linked to more efficient mastery of the material (Cooper, 1989). In this way, instructional quality in class and task quality in homework represent two quality-related dimensions that are relevant to students' learning experiences. Taken together, Carroll's model implies that students' learning can be understood in relation to both time and quality across two complementary contexts of classroom instruction and homework. Accordingly, four core learning inputs can be distinguished, including instructional time, instructional quality, homework time, and homework quality.

Most applications of Carroll's model have focused on academic achievement as the primary outcome. In a prospective review on this theoretical model of school learning, Carroll (1989) envisioned various expansions and adaptations of the model, and acknowledged Shulman's (1986) suggestion that the framework could be extended beyond cognitive processes and outcomes to add considerations of affective and motivational dimensions, for example, examining how these learning factors relate to students' learning interest. Available evidence has indicated that the influence of time and quality on students' learning interest may present different patterns from those observed for achievement. Specifically, high-quality learning tasks appears to stimulate students' interest, whereas prolonged time investment may be linked to lower interest (Shao et al., 2024; Xu et al., 2021). Therefore, understanding how time and quality affect not only achievement but also interest, across both instructional and homework contexts, remains an important yet underexplored issue.

### 2.2 The role of instructional time, instructional clarity, homework time, and homework quality in mathematics achievement

#### 2.2.1 Mathematics instructional time and instructional clarity

Instructional time is widely recognized as a primary predictor of mathematics achievement (Aksoy and Link, 2000; Cattaneo et al., 2017; Lavy, 2020). Policymakers aiming to improve students' achievement often seek to increase their school-related instructional time (OECD, 2004), as its positive effect exceeds other input indices, such as per-pupil expenditure, class size, or teacher training programs (Coates, 2003; Dobbie and Fryer, 2013; Mandel et al., 2019). However, a ceiling effect was also observed that excessive instructional time might be accompanied by physical and mental fatigue, which could be reflected in diminished returns or even lower achievement (Yang and Zhao, 2021; Yeşil Dagli, 2018). Therefore, researchers are inclined to support the complex nonlinear relationship between mathematics instructional time and achievement (Connolly, 2021).

Instead of simply increasing time, researchers proposed that students can perform better when they perceive higher quality of instructional (Rivkin and Schiman, 2015; Trautwein and Köller, 2003). In Carroll's model, the characteristics of high-quality instruction have not been specifically defined, but it has been mentioned that students need to be clearly informed about what they are to learn and how they are to learn it. Students need to be adequately exposed to the learning materials, and the learning tasks are expected to be carefully sequenced and presented in a well-structured way (Carroll, 1963, 1989). In the present study, we draw on the instructional framework for mathematics specified by Kunter and Baumert (2006) and operationalize instructional quality through the aspect of instructional clarity.

Instructional clarity represents the extent to which teachers deliver explicit objectives and well-structured lessons (Klieme and Nilsen, 2022). Previous studies suggested that higher instructional clarity was positively related to students' understanding, classroom engagement, and mathematics achievement (Chen, 2023; Chen and Lu, 2022; Scherer and Nilsen, 2016). Crucially, instructional clarity may moderate the effectiveness of time. Previous theories suggested that the relationship between time and achievement is heterogeneous (Scheerens, 2013), and the benefits of instructional time are likely amplified when learning content is conveyed in a structured and clear manner (Motegi and Oikawa, 2019).

#### 2.2.2 Mathematics homework time and homework quality

In the meta-analysis conducted by Cooper et al. (2006), homework time was found to be positively related to academic achievement, with an average correlation coefficient of 0.24. Mathematics homework extends the time that students allocate to mathematics learning, which is related to higher mathematics achievement (Dettmers et al., 2009; Trautwein et al., 2002). Despite these academic benefits, excessive homework may become counterproductive due to academic overload, which may impose substantial academic burdens and negatively affect students' performance in mathematics (Carroll, 1989; Cooper et al., 2006). A nonlinear relationship between mathematics homework time and academic outcomes has been revealed by quadratic regression analyses (Chin et al., 2022; Shao et al., 2024).

The quality of homework serves as a key indicator of out-of-class educational effectiveness. Well-selected, curriculum-aligned tasks can increase engagement, thereby positively predicting achievement (Rosário et al., 2018). Moreover, high-quality homework may compensate for insufficient homework time. Xu (2022) revealed that students who spent 30 min on high-quality homework achieved results similar to those spent 110 min on lower-quality tasks. Other empirical research examined how homework quality factors moderate the nonlinear relationship between homework time and learning outcomes. Yang and Zhao (2021) compared the achievement curves between rural and urban students, with the latter generally receiving higher quality of homework. They found that for urban students, learning for 3 h produced the same outcomes as learning for 4 h did for rural students. Furthermore, improving quality factors like self-regulated learning can also advance the threshold at which students achieved optimal mathematics achievement (Qin and Wan, 2025).

### 2.3 The role of instructional time, instructional clarity, homework time, and homework quality in mathematics interest

#### 2.3.1 Mathematics instructional time and instructional clarity

The impact of instructional time on students' mathematics interest remains mixed. Some studies demonstrated the positive associations between instructional time and students' attitudes toward mathematics (Meroni and Abbiati, 2016; Niki, 2024; Wedel, 2021). Students' mathematics interest might be higher when expanded mathematics lessons are accompanied by greater curiosity and engagement (Niki, 2024). In contrast, other studies indicated that with the extension of instructional time, there was a significantly increase in students perceived psychological effort costs and a decline in their intrinsic interest in mathematics (Benden and Lauermann, 2022; Carroll, 1989).

Instruction with high clarity is characterized by lower ambiguity and clearer organization of learning content, and has been consistently linked to positive motivational outcomes (Maulana et al., 2016; Scherer and Nilsen, 2016). Based on the Carroll model, it is rational to speculate that instructional clarity acts as a safeguard, that is, the positive association between instructional time and interest may be strengthened when students are clearly informed about their learning expectations and the well-structured learning contents.

#### 2.3.2 Mathematics homework time and homework quality

Currently, how mathematics homework time affects students' mathematics interest remains unclear. Limited evidence suggested that increasing homework time with attractive approaches encourages students to be more involved in mathematics activities and increases their interest (Young et al., 2017). However, overlearning also carries a high risk of diminishing mathematics interest (Cooper et al., 2006).

Homework quality may provide a useful perspective for understanding why homework time is not always linked to students' mathematics interest in the same way. Teachers' attentiveness to students' needs and interests when designing tasks is key to maintaining motivation (Rosário et al., 2019). High-quality homework characterized by engaging and personalized tasks may be linked to fewer negative emotions during the homework process, which may contribute to students' mathematics interest (Xu et al., 2021). Thus, the interaction between time and quality is essential for understanding how out-of-class activities influence the motivational dimensions of mathematics learning.

### 2.4 The present study

Carroll's model of school learning has been widely applied in the research field of mathematics education to examine how instructional time and instructional quality are related to students' academic outcomes. Some studies have further extended this framework to the context of homework by considering both homework time and homework quality. However, prior research has primarily focused on mathematics achievement, whereas considerably less attention has been paid to motivational outcomes such as mathematics interest. In addition, relatively few studies have simultaneously examined how time and quality factors interact within both classroom instruction and homework contexts in relation to students' mathematics achievement and mathematics interest.

To fill in these research gaps, the primary goals of this study are to delineate the contributions of time vs. quality, as well as their interactive effects on students' mathematics achievement and mathematics interest in the context of in-class instruction and out-of-class homework, using a sample of Chinese students in eighth grade.

Two research questions are proposed as follows.

(1) How much do instructional time, instructional clarity, homework time, and homework quality contribute to explaining the variance in mathematics achievement? And how these variables related to mathematics achievement?
(2) How much do instructional time, instructional clarity, homework time, and homework quality contribute to explaining the variance in mathematics interest? And how these variables related to mathematics interest?

## 3 Materials and methods

### 3.1 Participants

Participants were recruited from a city in South China comprising nine counties. The study employed a multistage sampling procedure following the Programme for International Student Assessment (PISA; OECD, 2024). First, the number of schools sampled from each county was determined proportionally based on the total number of schools in that county. Within each county, schools were then sampled using probabilities proportional to size (PPS), such that schools with larger student enrollments had a higher probability of selection. After that, approximately 35 eighth-grade students were randomly selected from each sampled school. The final sample consisted of 2,808 eighth-grade students (45.5% female) from 76 schools across the nine counties.

The study was approved by the ethics committee of the authors' host institution under approval number 2021–28. Informed consent was obtained from the principals, parents, and students at the participating schools. All the participating students were informed of the voluntary and anonymous nature of the study. They could withdraw from the study at any time at their or their parents' discretion.

### 3.2 Measures

#### 3.2.1 Mathematics achievement

The mathematics achievement test contained 35 multiple-choice items involving content strands of numbers and algebra, space and shape, and statistics and uncertainty. A correct response to each item counted for 1 point, and an incorrect response counted for 0 point. The internal consistency of the test was 0.90, and the average item difficulty coefficient was 0.70. A unidimensional Rasch model was estimated in R 4.2.2 to calibrate item difficulty and student ability parameters. The mean item difficulty was fixed at 0 to identify the scale. Item parameters were estimated using marginal maximum likelihood estimation (MML), and students' mathematics achievement scores were estimated using Expected a Posteriori (EAP) estimation. The resulting achievement scores were standardized with a mean of 500 and a standard deviation of 100.

#### 3.2.2 Mathematics interest

The mathematics interest scale was adapted from the PISA 2012 (OECD, 2013). The scale consisted of four items (e.g., “I do mathematics because I enjoy it”) measuring students' feelings about learning mathematics on a 4-point Likert scale ranging from 1 (strongly disagree) to 4 (strongly agree). The internal consistency of this scale was 0.950. The internal structure validity was examined using a unidimensional generalized partial credit model (GPCM). Item parameters were estimated using MML. Indices of root-mean square error of approximation (RMSEA), comparative fit index (CFI), and Tucker-Lewis index (TLI) were used to evaluate model fit. The model showed good fit to the data (RMSEA = 0.023; CFI = 0.994; TLI = 0.987). The scale score was operationalized as the mean score across the four items, with higher scores indicating higher mathematics interest.

#### 3.2.3 Mathematics instructional time (IT)

Mathematics instructional time was measured by the following question: “On average, how many mathematics lessons do you take each week?” Students' responses ranged from 0 to 15.

#### 3.2.4 Mathematics instructional clarity (IC)

The instructional clarity scale was adapted from TIMSS 2019 (Mullis and Martin, 2017). The scale consisted of seven items (e.g., “I know what my teacher expects me to do”) measuring students' feelings about mathematics class on a four-point Likert scale ranging from 1 (strongly disagree) to 4 (strongly agree). The internal consistency of this scale was 0.917. GPCM results showed good fit to the data (RMSEA = 0.005; CFI = 0.983; TLI = 0.984). Instructional clarity was operationalized as the mean score across the seven items, with higher scores indicating greater instructional clarity.

#### 3.2.5 Mathematics homework time (HT)

Mathematics homework time was measured by the following question: “On average, how much time do you typically spend each day on mathematics homework?” Students were asked to report their homework time based on the following options [1 = “no more than 15 min”; 2 = “15 to 30 min (including 30 min)”; 3 = “30 to 60 min (including 60 min)”; 4 = “60 to 90 min (including 90 min)”; 5 = “90 to 120 min (including 120 min)”; and 6 = “over 120 min”].

For easier interpretation and to maintain equal 30-min intervals across categories, response options 1 and 2 were combined into a single category representing “no more than 30 min” of homework time. Because only three students selected the “over 120 min” category, options 5 and 6 were merged into a single category representing “over 90 min” of homework time. Consequently, mathematics homework time was classified into four categories (HT1 “no more than 30 min,” HT2 “30–60 min,” HT3 “60–90 min,” and HT4 “over 90 min”). Three dummy variables were created to represent the categorical homework-time variable, with the HT1 group serving as the reference category.

#### 3.2.6 Mathematics homework quality (HQ)

The homework quality scale was adapted from Trautwein et al. (2006). The scale consisted of eight items (e.g., “Mathematics homework makes me think”) measuring students' feelings about their mathematics homework on a 4-point Likert scale ranging from 1 (strongly disagree) to 4 (strongly agree). The internal consistency of this scale was 0.907. GPCM results showed good fit to the data (RMSEA = 0.027; CFI = 0.963; TLI = 0.981). Homework quality was operationalized as the mean score across the eight items, with higher scores indicating higher homework quality.

#### 3.2.7 Covariates

As students' learning outcomes might be largely influenced by background variables, information about students' sex (0 = male/1 = female) and socioeconomic status (SES) was also collected as covariates.

### 3.3 Data analysis

Missing rates for the main variables ranged from 0 to 0.2%. Missing data were handled using Full Information Maximum Likelihood (FIML) estimation in Mplus 8.3 [Muthén & Muthén, Los Angeles, CA, USA (https://www.statmodel.com/)], which used all available information from incomplete cases. To examine the effects of instructional and homework-related learning inputs on students' mathematics achievement and mathematics interest, a series of regression models were conducted. The robust maximum likelihood estimator (MLR) was adopted to obtain parameter estimates and standard errors.

The data had a hierarchical structure in which students were nested within schools, and schools were further nested within counties. Because only nine counties were included in the sample, fitting a three-level model would substantially increase model complexity and reduce interpretability. Therefore, a two-level model was adopted.

The intraclass correlation coefficient (ICC) for mathematics achievement was 0.148, indicating that 14.8% of the variance in mathematics achievement was attributable to between-school difference. Therefore, multilevel regression models were estimated to account for the nested structure of the data and to examine both within-school and between-school effects on mathematics achievement.

For mathematics interest, the ICC was 0.035 and was lower than the commonly used threshold of 0.05 (Hayes, 2006). Nevertheless, given the nested structure of the data, a two-level random-intercept model was estimated. The analyses showed that the residual between-school variance was 0.005 and statistically non-significant after accounting for the random intercept. To account for potential clustering while maintaining a parsimonious model, the between-school level of the mathematics interest model was restricted to the random intercept, and no additional school-level predictors were included.

#### 3.3.1 The multilevel regression model for mathematics achievement

Variables at the within-school level were aggregated to the between-school level by calculating school mean scores. To reduce multicollinearity, student-level variables were group-mean centered, and school-level variables were grand-mean centered.

As shown in Equation 2, students' SEX and SES were first entered as control variables at both the within-school and between-school levels in Model A0. In Model A1, the linear and quadratic terms of instructional time (IT and IT^2^) were added at both levels to examine whether the association between instructional time and mathematics achievement was linear or curvilinear. The linear term reflected the immediate rate of change in mathematics achievement, whereas the quadratic term captured the acceleration or deceleration of these changes. In Model A2, instructional clarity (IC) was added at both levels. Model A3 further examined the interaction effects between instructional time and instructional clarity (IT × IC and IT^2^ × IC) at both levels.

To extend the analyses to the homework context, homework time was represented by three dummy variables of HT2 (30–60 min), HT3 (60–90 min), and HT4 (over 90 min), with HT1 (lower than 30 min) serving as the reference category. These dummy variables were entered at both levels in Model A4. Homework quality (HQ) was then added at both levels in Model A5. Finally, Model A6 included the interaction terms between homework time and homework quality (HT2 × HQ, HT3 × HQ, HT4 × HQ) at both levels.


$$\begin{array}{l} \text{Mathematics }{\text{achievement}}_{ij}\\ =\gamma_{00}+\gamma_{01}SEX_{B,j}+\gamma_{02}SES_{B,j}+\gamma_{10}IT_{B,j}+\gamma_{20}IT_{B,j}^{2}+\gamma_{03}IC_{B,j}\\ +\gamma_{13}\left(IT_{B,j}\times IC_{B,j}\right)+\gamma_{23}\left(IT_{B,j}^{2}\times IC_{B,j}\right)+\gamma_{30}HT2_{B,j}\\ +\gamma_{40}HT3_{B,j}+\gamma_{50}HT4_{B,j}+\gamma_{04}HQ_{B,j}+\gamma_{34}\left(HT2_{B,j}\times HQ_{B,j}\right)\\ +\gamma_{44}\left(HT3_{B,j}\times HQ_{B,j}\right)+\gamma_{54}\left(HT4_{B,j}\times HQ_{B,j}\right)+\beta_{01}SEX_{W,ij}\\ +\beta_{02}SES_{W,ij}+\beta_{10}IT_{W,ij}+\beta_{20}IT_{W,ij}^{2}+\beta_{03}IC_{W,ij}\\ +\beta_{13}\left(IT_{W,ij}\times IC_{W,ij}\right)+\beta_{23}\left(IT_{W,ij}^{2}\times IC_{W,ij}\right)+\beta_{30}HT2_{W,ij}\\ +\beta_{40}HT3_{W,ij}+\beta_{50}HT4_{W,ij}+\beta_{04}HQ_{W,ij}\\ +\beta_{34}\left(HT2_{W,ij}\times HQ_{W,ij}\right)+\beta_{44}\left(HT3_{W,ij}\times HQ_{W,ij}\right)\\ +\beta_{54}\left(HT4_{W,ij}\times HQ_{W,ij}\right)+\mu_{0j}+r_{ij} \end{array}$$ (2)

In Equation 2, the subscripts *i* and *j* denote students and schools, respectively. The term μ_0*j*_ denotes the random effect at the between-school level, whereas *r*_*ij*_ represents the residual error at the within-school level. For each model, within-school and between-school coefficients of determination (*R*^2^ and adjusted *R*^2^) were estimated. The incremental contribution of each block of predictors was evaluated by examining changes in explained variance (*R*^2^ and adjusted *R*^2^) across successive models.

#### 3.3.2 The multilevel regression model for mathematics interest

Equation 3 presented the two-level random-intercept model predicting students' mathematics interest. After controlling for students' SEX and SES in Model B0, IT and IT^2^ were entered in Model B1, followed by IC in Model B2. The interaction effects between IT and IC were further examined in Model B3. In Model B4, three dummy variables representing HT were added. HQ was subsequently included in Model B5, and the interaction terms between HT and HQ were further entered in Model B6. Moreover, a random intercept (μ_0*j*_) was included to account for potential clustering effects.


$$\begin{array}{l} {\text{Mathematics interest}}_{ij}\\ =\gamma_{00}+\beta_{01}SEX_{ij}+\beta_{02}SES_{ij}+\beta_{10}IT_{ij}+\beta_{20}IT_{ij}^{2}+\beta_{03}IC_{ij}\\ +\beta_{13}\left(IT_{ij}\times IC_{ij}\right)+\beta_{23}\left(IT_{ij}^{2}\times IC_{ij}\right)+\beta_{30}{HT2}_{ij}+\beta_{40}{HT3}_{ij}\\ +\beta_{50}{HT4}_{ij}+\beta_{04}HQ_{ij}+\beta_{34}\left({HT2}_{ij}\times HQ_{ij}\right)\\ +\beta_{44}\left({HT3}_{ij}\times HQ_{ij}\right)+\beta_{54}\left({HT4}_{ij}\times HQ_{ij}\right)+\mu_{0j}+r_{ij} \end{array}$$ (3)

To reduce multicollinearity, variables were mean centered. The *R*^2^, adjusted *R*^2^, *R*^2^ and adjusted *R*^2^ were estimated for each model.

## 4 Results

### 4.1 Common method bias test

To address the possible biases issue due to the reliance on self-reported measures, Harman's single-factor test was conducted. The results of the exploratory factor analysis revealed six factors with eigenvalues greater than one, with the first factor accounting for 38.56% of the variance, which is below the commonly used threshold of 40%. However, we need to acknowledge that Harman's single-factor test only provided a limited diagnostic assessment and cannot fully rule out common method bias.

### 4.2 Regression diagnostics

The results of multicollinearity diagnostics showed that all tolerance values were above 0.20 and all variance inflation factor (VIF) values were below 5, indicating no serious multicollinearity concerns (see Supplementary Table S1). The histograms of standardized residuals for mathematics achievement and mathematics interest were presented in Supplementary Figures S2, S3. The skewness values were −0.39 and −0.55 for mathematics achievement and mathematics interest, and the corresponding kurtosis values were −0.51 and −0.20, suggesting no serious violations of the normality assumption. In addition, all Cook's distance values were below 1, indicating that no single case exerted undue influence on the regression estimates.

### 4.3 The role of instructional time, instructional clarity, homework time, and homework quality in mathematics achievement

#### 4.3.1 Effects of instructional time and instructional clarity

The standardized regression coefficients and standard errors of main variables on mathematics achievement were presented in Table 1. The unstandardized coefficients and the 95% confidence intervals were presented in Supplementary Tables S2–S4.

**Table 1:** Standardized regression coefficients and standard errors for the multilevel regression models predicting mathematics achievement.

Variables	Model A0	Model A1	Model A2	Model A3	Model A4	Model A5	Model A6
Within-school level							
SEX_W_ (β_01_)	−0.10 (0.02)^***^	−0.10 (0.02)^***^	−0.10 (0.02)^***^	−0.10 (0.02)^***^	−0.10 (0.02)^***^	−0.10 (0.02)^***^	−0.10 (0.02)^***^
SES_W_ (β_02_)	0.20 (0.03)^***^	0.19 (0.03)^***^	0.17 (0.02)^***^	0.17 (0.02)^***^	0.17 (0.02)^***^	0.17 (0.02)^***^	0.17 (0.02)^***^
IT_W_ (β_10_)		0.09 (0.03)^**^	0.09 (0.03)^***^	0.09 (0.03)^**^	0.09 (0.03)^**^	0.09 (0.03)^**^	0.09 (0.03)^**^
${\text{IT}}_{\text{W}}^{2}$ (β_20_)		−0.07 (0.03)^*^	−0.06 (0.03)^*^	−0.07 (0.03)^*^	−0.06 (0.03)^*^	−0.06 (0.03)^*^	−0.06 (0.03)^*^
IC_W_ (β_03_)			0.17 (0.02)^**^	0.18 (0.02)^***^	0.19 (0.02)^***^	0.19 (0.02)^***^	0.19 (0.02)^***^
IT_W_×*IC*_W_ (β_13_)				−0.01 (0.02)	−0.00 (0.02)	−0.00 (0.02)	0.00 (0.02)
${\text{IT}}_{\text{W}}^{2}\times IC_{\text{W}}$ (β_23_)				−0.02 (0.02)	−0.02 (0.02)	−0.02 (0.02)	−0.01 (0.02)
HT2_W_ (β_30_)					0.06 (0.02)^**^	0.06 (0.02)^**^	0.06 (0.02)^**^
HT3_W_ (β_40_)					0.01 (0.02)	0.01 (0.02)	0.01 (0.02)
HT4_W_ (β_50_)					0.01 (0.02)	0.01 (0.02)	0.01 (0.02)
HQ_W_ (β_04_)						−0.01 (0.02)	0.03 (0.03)
HT2_W_×*HQ*_W_ (β_34_)							−0.03 (0.02)
HT3_W_×*HQ*_W_ (β_44_)							−0.05 (0.02)^**^
HT4_W_×*HQ*_W_ (β_54_)							−0.04 (0.02)
Between-school level							
Intercept_B_ (γ_00_)	21.40 (3.86)^***^	21.97 (3.85)^***^	20.70 (3.49)^***^	20.56 (3.41)^***^	20.31 (3.28)^***^	21.00 (3.32)^***^	20.47 (3.13)^***^
Sex_B_ (γ_01_)	−0.01 (0.11)	0.00 (0.10)	0.01 (0.10)	0.02 (0.10)	0.03 (0.11)	−0.02 (0.12)	0.01 (0.12)
SES_B_ (γ_02_)	0.81 (0.08)^***^	0.82 (0.08)^***^	0.83 (0.08)^***^	0.78 (0.09)^***^	0.82 (0.08)^***^	0.79 (0.09)^***^	0.76 (0.09)^***^
IT_B_ (γ_10_)		0.06 (0.14)	0.03 (0.13)	−0.06 (0.12)	−0.16 (0.14)	−0.17 (0.13)	−0.17 (0.13)
${\text{IT}}_{\text{B}}^{2}$ (γ_20_)		−0.02 (0.12)	−0.01 (0.11)	0.14 (0.14)	0.24 (0.13)	0.27 (0.12)^*^	0.24 (0.12)
IC_B_ (γ_03_)			−0.19 (0.10)	−0.10 (0.12)	−0.07 (0.12)	0.12 (0.19)	0.11 (0.18)
IT_B_ · *IC*_B_ (γ_13_)				0.16 (0.12)	0.16 (0.12)	0.14 (0.12)	0.08 (0.12)
${\text{IT}}_{\text{B}}^{2}$ · *IC*_B_ (γ_23_)				−0.24 (0.18)	−0.36 (0.19)	−0.40 (0.18)^*^	−0.38 (0.18)^*^
HT2_B_ (γ_30_)					0.16 (0.10)	0.13 (0.11)	0.13 (0.10)
HT3_B_ (γ_40_)					−0.14 (0.08)	−0.12 (0.08)	−0.11 (0.08)
HT4_B_ (γ_50_)					−0.06 (0.11)	−0.07 (0.11)	−0.05 (0.11)
HQ_B_ (γ_04_)						−0.22 (0.17)	−0.22 (0.29)
HT2_B_×*HQ*_B_ (γ_34_)							0.44 (0.24)
HT3_B_×*HQ*_B_ (γ_44_)							0.12 (0.12)
HT4_B_×*HQ*_B_ (γ_54_)							−0.00 (0.02)
Within *R*^2^ (Adj. *R*^2^)	0.031 (0.030)	0.036 (0.035)	0.065 (0.063)	0.065 (0.063)	0.068 (0.065)	0.068 (0.065)	0.071 (0.067)
Within Δ*R*^2^ (Adj. Δ*R*^2^)	0.031 (0.030)	0.005 (0.005)	0.029 (0.028)	0.000 (0.000)	0.003 (0.002)	0.000 (0.000)	0.003 (0.002)
Between *R*^2^ (Adj. *R*^2^)	0.833 (0.829)	0.857 (0.849)	0.857 (0.849)	0.867 (0.854)	0.891 (0.874)	0.896 (0.878)	0.908 (0.887)
Between Δ*R*^2^ (Adj. Δ*R*^2^)	0.833 (0.829)	0.024 (0.020)	0.000 (0.000)	0.010 (0.005)	0.024 (0.020)	0.005 (0.004)	0.012 (0.009)

^*^p < 0.05, ^**^p < 0.01, ^***^p < 0.001. The values presented in the tables are standardized regression coefficients. HT2–HT4 are dummy-coded variables for homework time categories, with HT1 as the reference category.

After controlling for students' sex and SES at both the within-school and between-school levels, instructional time explained an additional 0.5% of the within-school variance and 2.4% of the between-school variance in mathematics achievement (Model A1). The inclusion of instructional clarity in Model A2 further increased the explained variance by 2.9% at the within-school level, whereas the between-school level explained variance remained unchanged.

**Figure 1:**
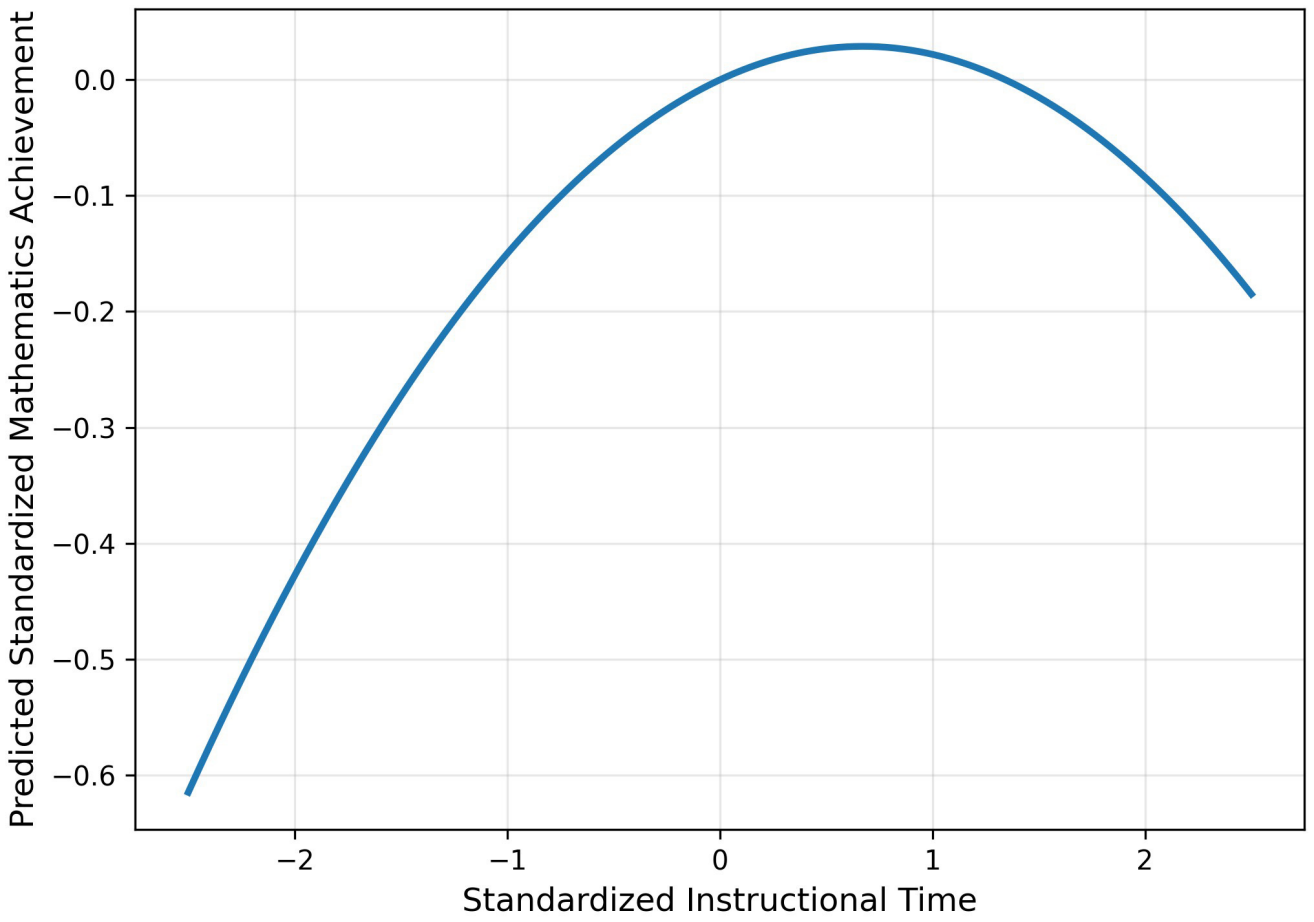
The nonlinear relationship between instructional time and mathematics achievement at the within-school level.

The results of Model A6 showed that there was no significant interaction between instructional time and instructional clarity at the within-school level (β_13_ = 0.00, *p*>0.05; β_23_ = −0.01, *p*>0.05). Instructional clarity positively predicted students' mathematics achievement at the within-school level (β_03_ = 0.19, *p* < 0.001). Regardless of instructional clarity, as Figure 1 showed, the trajectories of mathematics achievement exhibited a significant initial increase with rising instructional time (β_10_ = 0.09, *p* < 0.01), followed by a pattern of deceleration (β_20_ = −0.06, *p* < 0.05), indicating a curvilinear relationship between instructional time and achievement.

At the between-school level, although the interaction between the quadratic term of instructional time and instructional clarity was significant in Model A6 (γ_23_ = −0.38, *p* < 0.05), the non-significant linear effects and lower-order interaction terms suggested that the practical interpretation of this higher-order interaction was limited.

#### 4.3.2 Effects of homework time and homework quality

As the results of Model A4 showed, homework time explained an additional 0.3% of the within-school variance and 2.4% of the between-school in mathematics achievement (Model A4). The inclusion of homework quality in Model A5 did not substantially increase the explained variance at the within-school level but contributed an additional 0.5% of the explained variance at the between-school level.

**Figure 2:**
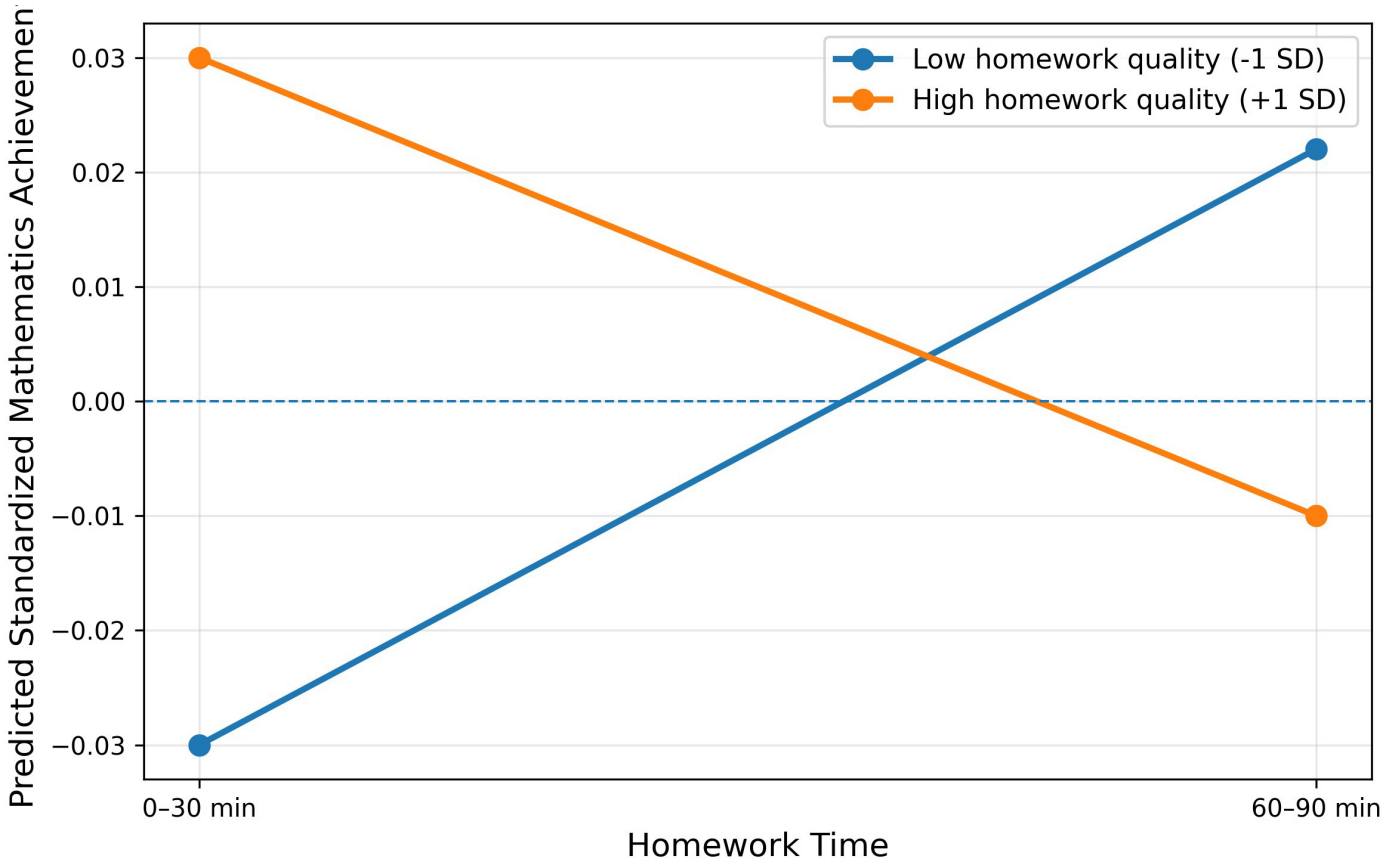
The interaction effect between homework time and homework quality on mathematics achievement at the within-school level.

The results of Model A6 indicated that homework quality was negatively interacted with the term of *HT*3_*W*_ at the within-school level (β_44_ = −0.05, *p* < 0.01). Specifically, compared with students who spent no more than 30 min on homework (HT1, the reference group), the association between spending 60–90 min on homework (HT3) and mathematics achievement became weaker as perceived homework quality increased. As the simple slope test shown in Figure 2, among students reporting lower homework quality, spending 60–90 min on homework was associated with higher mathematics achievement than spending no more than 30 min on homework. However, as homework quality increased, the achievement gap among students spending different amounts of time on homework narrowed. Under conditions of higher homework quality, spending more time on homework was even negatively associated with mathematics achievement.

### 4.4 The role of instructional time, instructional clarity, homework time, and homework quality in mathematics interest

#### 4.4.1 Effects of instructional time and instructional clarity

The standardized regression coefficients and standard errors of main variables on mathematics interest were presented in Table 2. The unstandardized coefficients and the 95% confidence intervals were presented in Supplementary Tables S5–S7.

**Table 2:** Standardized regression coefficients and standard errors for the multilevel regression models predicting mathematics interest.

Variables	Model B0	Model B1	Model B2	Model B3	Model B4	Model B5	Model B6
Within-school level							
SEX (β_01_)	−0.14 (0.02)^***^	−0.14 (0.02)^***^	−0.14 (0.01)^***^	−0.14 (0.01)^***^	−0.14 (0.01)^***^	−0.13 (0.01)^***^	−0.13 (0.01)^***^
SES (β_02_)	0.09 (0.03)^***^	0.10 (0.03)^***^	0.03 (0.02)	0.03 (0.02)	0.03 (0.02)	0.02 (0.02)	0.02 (0.02)
IT (β_10_)		−0.07 (0.03)^*^	−0.04 (0.03)	−0.04 (0.03)	−0.04 (0.03)	−0.03 (0.02)	−0.03 (0.02)
IT^2^ (β_20_)		0.01 (0.02)	0.02 (0.02)	0.02 (0.02)	0.03 (0.02)	0.02 (0.01)	0.02 (0.01)
IC (β_03_)			0.55 (0.02)^***^	0.56 (0.02)^***^	0.55 (0.02)^***^	0.32 (0.03)^***^	0.32 (0.03)^***^
IT × *IC* (β_13_)				0.05 (0.02)^*^	0.05 (0.02)^*^	0.04 (0.02)^*^	0.05 (0.02)^*^
IT^2^×*IC* (β_23_)				−0.02 (0.02)	−0.02 (0.02)	−0.02 (0.02)	−0.02 (0.02)
HT2 (β_30_)					−0.02 (0.02)	−0.01 (0.02)	−0.01 (0.02)
HT3 (β_40_)					−0.04 (0.02)	−0.03 (0.02)	−0.03 (0.02)
HT4 (β_50_)					−0.05 (0.02)^*^	−0.04 (0.02)	−0.04 (0.02)
HQ (β_04_)						0.38 (0.03)^***^	0.41 (0.03)^***^
HT2 × *HQ* (β_34_)							−0.04 (0.02)
HT3 × *HQ* (β_44_)							−0.03 (0.02)
HT4 × *HQ* (β_54_)							−0.00 (0.02)
Between-school level							
Intercept_B_ (γ_00_)	19.50 (2.77)^***^	20.20 (2.73)^***^	35.66 (7.76)^***^	35.34 (7.49)^***^	37.00 (7.82)^***^	44.75 (10.12)^***^	45.17 (10.49)^***^
*R*^2^ (Adjusted*R*^2^)	0.019 (0.019)	0.023 (0.022)	0.327 (0.326)	0.329 (0.328)	0.333 (0.331)	0.417 (0.415)	0.418 (0.415)
Δ*R*^2^ (AdjustedΔ*R*^2^)	0.019 (0.019)	0.004 (0.003)	0.304 (0.304)	0.002 (0.002)	0.004 (0.003)	0.084 (0.084)	0.001 (0.000)

^*^p < 0.05, ^***^p < 0.001. The values presented in the tables are standardized regression coefficients. HT2–HT4 are dummy-coded variables for homework time categories, with HT1 as the reference category.

The explained variance in mathematics interest remained unchanged after instructional time was added in Model B1. When instructional clarity was added to Model B2, the explained variance increased by 30.4%.

**Figure 3:**
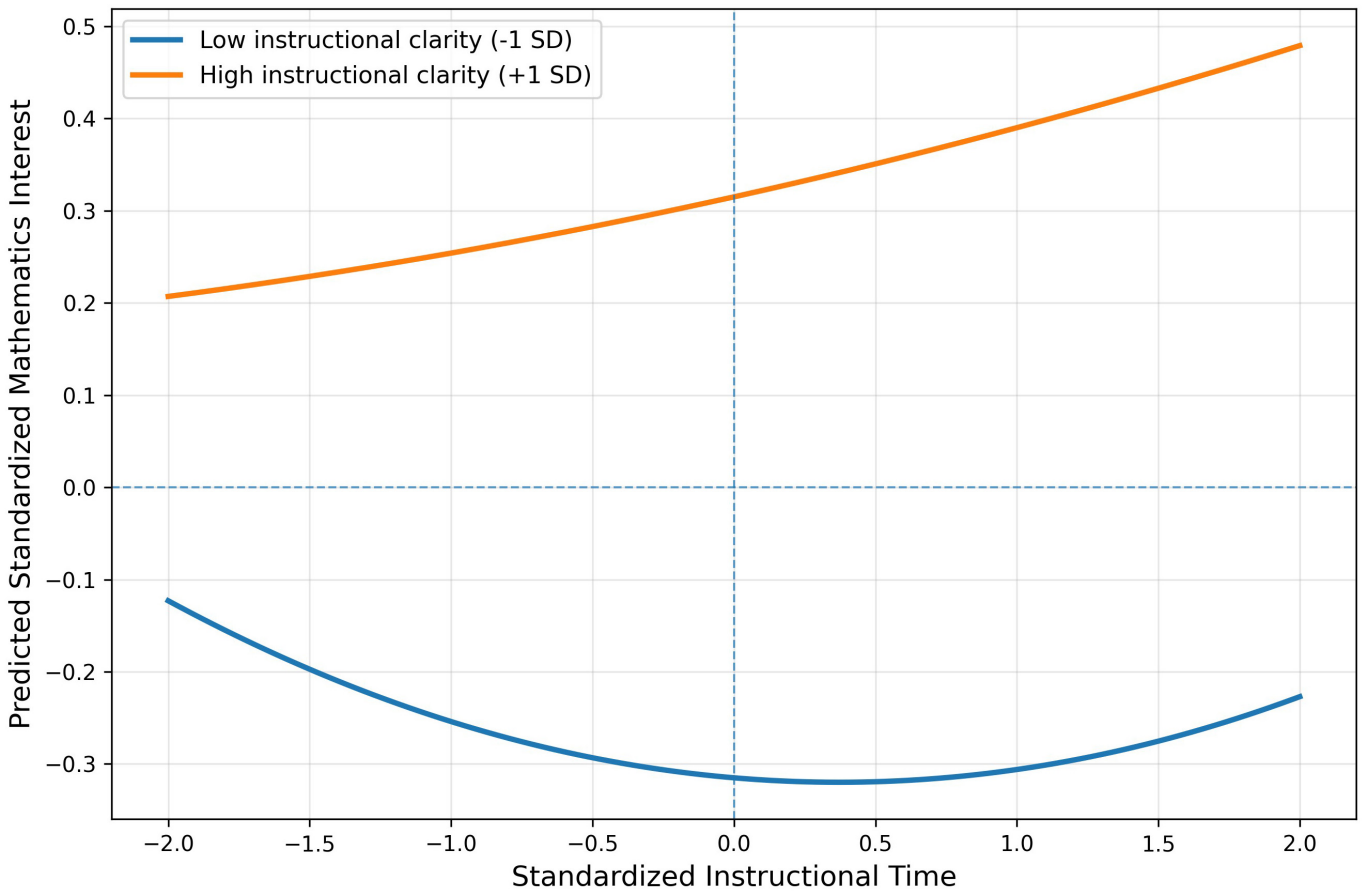
The interaction effect between instructional time and instructional clarity on mathematics interest.

According to the results of Model B6 in Table 2, the linear term of instructional time significantly interacted with instructional clarity in predicting mathematics interest (β_13_ = 0.05, *p* < 0.05). As Figure 3 showed, for students reporting higher levels of instructional clarity, their mathematics interests were strongly associated with instructional time.

#### 4.4.2 Effects of homework time and homework quality

Homework time did not account for any variance in mathematics interest (Model B4), while homework quality explained an additional 8.4% variance (Model B5).

There was no interactive effect between homework time and homework quality on students' mathematics interest (β_34_ = −0.04, *p*>0.05; β_44_ = −0.03, *p*>0.05; β_54_ = −0.00, *p*>0.05). Students' mathematics interest was positively associated with their mathematics homework quality (β_04_ = 0.41, *p* < 0.001).

## 5 Discussion

This study examined how instructional time, instructional clarity, homework time, and homework quality were associated with students' mathematics achievement and mathematics interest. Given the large sample size, some statistically significant associations explained only a very small proportion of additional variance, which should be interpreted cautiously. Therefore, the discussion focused primarily on findings with relatively stronger explanatory power and clearer educational relevance.

### 5.1 The role of instructional time, instructional clarity, homework time, and homework quality in mathematics achievement

For mathematics achievement, although the curvilinear effect of instructional time and the interaction between homework time and homework quality were statistically significant, they explained only 0%−0.5% variance in achievement and therefore provided limited educational implications. Instructional clarity accounted for 2.9% of the variance at the within-school level and showed the most practical significance.

This result was consistent with prior studies emphasizing instructional clarity as an important feature of effective teaching (Hattie, 2023; Renninger and Hidi, 2019). Clear instruction helps students understand learning goals, task requirements, and the logical relations among mathematical ideas. It is related to greater accessibility of learning materials and lower unnecessary cognitive demands (Klieme and Nilsen, 2022). From an educational perspective, this finding suggested that improving the clarity and organization of classroom instruction may be more meaningful than simply increasing instructional or homework time.

At the between-school level, although the interaction between the quadratic term of instructional time and instructional clarity reached statistical significance, its effect size was small. In addition, the nonsignificant linear effects and lower-order interaction terms made the practical meaning of this interaction difficult to interpret. Therefore, these school-level findings should be regarded mainly as contextual information and should not be taken as strong evidence that school-level time allocation has substantial educational effects.

Overall, the findings supported previous arguments that challenged the assumption of “more time leads to better achievement” (Yang and Zhao, 2021). Instead, instructional clarity at the within-school level emerged as the predictor with the most practical significance, suggesting that the quality and organization of classroom instruction may be more closely related to achievement than the quantity of learning time.

### 5.2 The role of instructional time, instructional clarity, homework time, and homework quality in mathematics interest

For mathematics interest, although the interaction between instructional time and instructional clarity reached statistical significance, its effect size was negligible in practical terms. By contrast, instructional clarity was positively associated with mathematics interest and explained over 30% of its variance. From the perspective of self-determination theory, clear instruction was associated with students' sense of competence by helping them understand what they are expected to learn and how they can make progress (Deci and Ryan, 2002; Niemiec and Ryan, 2009). When instruction was clear and well-structured, students were more likely to experience mathematics learning as manageable and meaningful, which supported their interest in the subject.

Homework quality was independently associated with mathematics interest and accounted for more than 8% of its variance. However, homework time was not associated with students' mathematics interest. From the perspective of educational practice, longer homework time may reflect both greater engagement in mathematics learning and greater perceived burden or academic pressure, with these positive and negative aspects potentially offsetting each other (Cooper et al., 2006). In contrast, high-quality homework, characterized by appropriate challenge, meaningful practice, and clear relevance to learning goals, may be more closely linked to students' sense of purpose and accomplishment (Rosário et al., 2018; Xu, 2022).

Overall, these findings revealed the variabilities in the relationships between time and learning outcomes, supporting Godwin, Seltman, Almeda, Davis Skerbetz, Kai and Baker (2021) speculation that the effect of time varied depending on the measured time variables and outcome variables, the nature of learning activities, and the quality of learning experiences. By clarifying how time and quality interacted in both instructional and homework contexts, this study helped reconcile inconsistent findings in prior research on learning time and highlighted the central role of task quality in influencing whether time investment translated into achievement gains or sustained learning interest.

### 5.3 Implications and limitations

This study provides several implications for mathematics teaching and homework design. First, instructional clarity deserves particular attention. Among the predictors examined, instructional clarity showed the clearest practical relevance for mathematics achievement and was also positively associated with mathematics interest. Therefore, teachers may support students' mathematics learning by providing clearer explanations, more coherent task sequences, and better-structured classroom activities (Seidel et al., 2005). Moreover, homework quality appeared to be more relevant to mathematics interest than homework time. Rather than assigning more homework, teachers may consider whether homework tasks are meaningful, appropriately challenging, clearly connected to learning goals, and manageable for students (Rosário et al., 2019; Trautwein et al., 2006).

Compared with quality-related factors, increasing learning time alone may not be closely associated with better mathematics outcomes. Several time-related effects reached statistical significance but were small in effect sizes. Therefore, educational practice should avoid equating more time with better learning. Instead, greater attention should be given to whether instructional and homework time is used efficiently and experienced positively by students.

Several limitations of this study need to be acknowledged. First, this study was based on cross-sectional data, which does not allow us to establish temporal ordering among these variables. The use of longitudinal data that includes students' prior learning outcomes may allow for causal inference. Second, although students' sex and SES were controlled, other potentially relevant covariates were not included in the analyses. The omission of these variables may lead to residual confounding and limits the extent to which the observed associations can be interpreted as independent effects. Future research should consider incorporating a broader set of covariates to more fully account for individual and contextual differences in mathematics learning. Third, the data collected in this study were mostly self-reported by students. The use of a single data source may introduce bias arising from social desirability, shared response tendencies, or students' general perceptions of their mathematics learning experiences. Therefore, the observed relationships should be interpreted with appropriate caution. Future studies could incorporate multiple data sources, such as teacher reports, classroom observations, learning logs, or interviews, to triangulate students' self-reported data and provide a more comprehensive assessment of instructional and homework-related experiences.

## 6 Conclusions

In mathematics learning, the quality-related factors showed the most practical significance for its positive relationship with both mathematics achievement and mathematics interest. However, the effects of time-related variables and the interactions between time and quality showed limited practical significance. These findings suggest that improving the clarity of mathematics instruction and the quality of homework may be more educationally meaningful than simply increasing instructional or homework time.

## Data Availability

The raw data supporting the conclusions of this article will be made available by the authors, without undue reservation.
