# Supplementary material for: Time and quality in and out of class: the roles of instruction and homework in mathematics achievement and interest
Source: Front Psychol. 2026 Jun 23;17:1870861. doi: 10.3389/fpsyg.2026.1870861 (PMC13337771; doi:10.3389/fpsyg.2026.1870861)
Supplement: Supplementary file 1 [file Supplementary_file_1.docx]

# **Table S1**

**The results of collinearity diagnostics**

|  | Tolerance | VIF |
| --- | --- | --- |
| SEX | 0.990 | 1.010 |
| SES | 0.983 | 1.017 |
| IT | 0.818 | 1.222 |
| IT^2^ | 0.831 | 1.203 |
| IC | 0.514 | 1.947 |
| IT$\cdot IC$ | 0.750 | 1.333 |
| IT^2^$\cdot\mathrm{IC}$ | 0.616 | 1.625 |
| HT2 | 0.933 | 1.072 |
| HT3 | 0.929 | 1.076 |
| HT4 | 0.909 | 1.100 |
| HQ | 0.407 | 2.456 |
| HT2$\cdot HQ$ | 0.643 | 1.555 |
| HT3$\cdot\mathrm{HQ}$ | 0.858 | 1.166 |
| HT4$\cdot\mathrm{HQ}$ | 0.847 | 1.181 |

# **Table S2**

**Unstandardized regression coefficients and standard errors for the multilevel regression models predicting mathematics achievement**

| Variables | Model A0 | Model A1 | Model A2 | Model A3 | Model A4 | Model A5 | Model A6 |
| --- | --- | --- | --- | --- | --- | --- | --- |
| **Within-school level** |  |  |  |  |  |  |  |
| SEX_W_ ($B_{01}$) | –16.16 (3.08) ^***^ | –16.09 (3.06) ^***^ | –16.33 (3.06) ^***^ | –16.36 (3.06) ^***^ | –16.16 (3.04) ^***^ | –16.20 (3.08) ^***^ | –16.20 (3.09) ^***^ |
| SES_W_ ($B_{02}$) | 18.20 (2.40) ^***^ | 17.82 (2.40) ^***^ | 15.91 (2.28) ^***^ | 15.95 (2.29) ^***^ | 15.77 (2.29) ^***^ | 15.80 (2.30) ^***^ | 15.81 (2.28) ^***^ |
| IT_W_ ($B_{10}$) |  | 5.17 (1.50) ^**^ | 5.53 (1.58) ^***^ | 5.40 (1.58) ^**^ | 5.18 (1.54) ^**^ | 5.15 (1.55) ^**^ | 5.08 (1.51) ^**^ |
| IT^2^_W_ ($B_{20}$) |  | –1.30 (0.51) ^*^ | –1.27 (0.48) ^**^ | –1.28 (0.51) ^*^ | –1.26 (0.50) ^*^ | –1.24 (0.50) ^*^ | –1.25 (0.50) ^*^ |
| IC_W_ ($B_{03}$) |  |  | 27.75 (3.14) ^***^ | 28.80 (3.16) ^***^ | 29.63 (3.16) ^**^ | 30.34 (3.81) ^***^ | 30.18 (3.75) ^***^ |
| IT_W_$\times\mathrm{IC}$_W_ ($B_{13}$) |  |  |  | –0.54 (2.41) | –0.44 (2.44) | –0.40 (2.44) | 0.09 (2.47) |
| IT^2^_W_$\times\mathrm{IC}$_W_ ($B_{23}$) |  |  |  | –0.48 (0.63) | –0.52 (0.63) | –0.55 (0.62) | –0.43 (0.63) |
| HT2_W_ ($B_{30}$) |  |  |  |  | 10.11 (3.31) ^**^ | 10.10 (3.31) ^**^ | 10.17 (3.38) ^**^ |
| HT3_W_ ($B_{40}$) |  |  |  |  | 3.968 (7.94) | 3.91 (7.89) | 2.18 (7.97) |
| HT4_W_ ($B_{50}$) |  |  |  |  | 7.026 (10.79) | 6.91 (10.62) | 3.72 (10.87) |
| HQ_W_ ($B_{04}$) |  |  |  |  |  | –0.94 (3.42) | 4.21 (4.20) |
| HT2_W_$\times\mathrm{HQ}$_W_ ($B_{34}$) |  |  |  |  |  |  | –7.71 (5.92) |
| HT3_W_$\times\mathrm{HQ}$_W_ ($B_{44}$) |  |  |  |  |  |  | –23.48 (8.38) ^**^ |
| HT4_W_$\times\mathrm{HQ}$_W_ ($B_{54}$) |  |  |  |  |  |  | –21.85 (13.40) |
| **Between-school level** |  |  |  |  |  |  |  |
| Intercept_B_ ($Y_{00}$) | 523.74 (30.02) ^***^ | 524.00 (27.09) ^***^ | 521.39 (28.88) ^***^ | 518.38 (28.51) ^***^ | 505.65 (32.29) ^***^ | 522.00 (34.39) ^***^ | 513.87 (33.75) ^***^ |
| Sex_B_ ($Y_{01}$) | –7.34 (63.30) | 0.24 (56.88) | 5.12 (60.48) | 13.05 (60.43) | 17.49 (65.33) | –13.34 (70.30) | 7.20 (69.85) |
| SES_B_ ($Y$) | 32.03 (5.57) ^***^ | 31.61 (5.36) ^***^ | 33.57 (5.27) ^***^ | 31.76 (5.63) ^***^ | 33.02 (5.34) ^***^ | 31.61 (5.39) ^***^ | 30.60 (4.98) ^***^ |
| IT_B_ ($Y_{10}$) |  | 1.49 (3.67) | 0.85 (3.69) | –1.61 (3.46) | –4.46 (3.65) | –4.62 (3.50) | –4.82 (3.39) |
| IT^2^_B_ ($Y_{20}$) |  | –0.45 (2.29) | –0.24 (2.34) | 2.94 (2.83) | 5.00 (2.52) ^*^ | 5.46 (2.26) ^*^ | 4.90 (2.28) ^*^ |
| IC_B_ ($Y_{03}$) |  |  | –35.37 (17.92) ^*^ | –17.51 (22.58) | –12.74 (22.04) | 21.04 (33.45) | 19.36 (33.41) |
| IT_B_$\cdot IC$_B_ ($Y_{13}$) |  |  |  | 32.55 (24.17) | 31.33 (23.54) | 27.39 (22.99) | 15.24 (23.82) |
| IT^2^_B_$\cdot\mathrm{IC}$_B_ ($Y_{23}$) |  |  |  | –32.32 (22.66) | –47.33 (23.28) ^*^ | –52.00 (21.33) ^*^ | –50.40 (21.89) ^*^ |
| HT2_B_ ($Y_{30}$) |  |  |  |  | 32.80 (20.67) | 26.49 (21.19) | 26.02 (20.27) |
| HT3_B_ ($Y_{40}$) |  |  |  |  | –69.61 (39.19) | –60.53 (41.36) | –58.52 (37.74) |
| HT4_B_ ($Y_{50}$) |  |  |  |  | –36.21 (70.19) | –44.82 (73.62) | –33.59 (72.88) |
| HQ_B_ ($Y_{04}$) |  |  |  |  |  | –33.91 (25.77) | –34.32 (44.63) |
| HT2_B_$\times\mathrm{HQ}$_B_ ($Y_{34}$) |  |  |  |  |  |  | 224.03 (129.97) |
| HT3_B_$\times\mathrm{HQ}$_B_ ($Y_{44}$) |  |  |  |  |  |  | 259.53 (274.60) |
| HT4_B_$\times\mathrm{HQ}$_B_ ($Y_{54}$) |  |  |  |  |  |  | –3.04 (37.02) |

*Note.* ^*^*p* < 0.05, ^**^*p* < 0.01, ^***^*p* < 0.001. The values presented in the tables are unstandardized regression coefficients, with standard errors in parentheses. HT2–HT4 are dummy-coded variables for homework time categories (HT1 was used as the reference category).

# **Table S3**

**The 95% confidence intervals of unstandardized regression coefficients for the multilevel regression models predicting mathematics achievement**

| Variables | Model A0 | Model A1 | Model A2 | Model A3 | Model A4 | Model A5 | Model A6 |
| --- | --- | --- | --- | --- | --- | --- | --- |
| **Within-school level** |  |  |  |  |  |  |  |
| SEX_W_ | [–22.20, –10.13] | [–22.10, –10.09] | [–22.33, –10.33] | [–22.37, –10.36] | [–22.12, –10.20] | [–22.24, –10.17] | [–22.25, –10.16] |
| SES_W_ | [13.49, 22.90] | [13.12, 22.52] | [11.44, 20.38] | [11.47, 20.43] | [11.28, 20.26] | [11.30, 20.31] | [11.34, 20.28] |
| IT_W_ |  | [2.23, 8.12] | [2.44, 8.62] | [2.32, 8.50] | [2.15, 8.20] | [2.12, 8.18] | [2.11, 8.04] |
| IT^2^_W_ |  | [–2.30, –0.29] | [–2.22, –0.32] | [–2.28, –0.27] | [–2.23, –0.28] | [–2.22, –0.26] | [–2.23, –0.26] |
| IC_W_ |  |  | [21.69, 33.90] | [22.60, 34.99] | [23.44, 35.83] | [22.89, 37.80] | [22.83, 37.53] |
| IT_W_$\times\mathrm{IC}$_W_ |  |  |  | [–5.28, 4.19] | [–5.21, 4.34] | [–5.19, 4.39] | [–4.75, 4.92] |
| IT^2^_W_$\times\mathrm{IC}$_W_ |  |  |  | [–1.71, 0.75] | [–1.75, 0.71] | [–1.76, 0.67] | [–1.66, 0.81] |
| HT2_W_ |  |  |  |  | [3.63, 16.60] | [3.61, 16.59] | [3.54, 16.79] |
| HT3_W_ |  |  |  |  | [–11.59, 19.53] | [–11.55, 19.36] | [–13.44, 17.79] |
| HT4_W_ |  |  |  |  | [–14.12, 28.18] | [–13.92, 27.73] | [–17.58, 25.02] |
| HQ_W_ |  |  |  |  |  | [–7.64, 5.75] | [–4.03, 12.45] |
| HT2_W_$\times\mathrm{HQ}$_W_ |  |  |  |  |  |  | [–19.31, 3.89] |
| HT3_W_$\times\mathrm{HQ}$_W_ |  |  |  |  |  |  | [–39.90, –7.06] |
| HT4_W_$\times\mathrm{HQ}$_W_ |  |  |  |  |  |  | [–48.10, 4.41] |
| **Between-school level** |  |  |  |  |  |  |  |
| Intercept_B_ | [464.89, 582.58] | [470.91, 577.09] | [464.78, 578.00] | [462.49, 574.267] | [442.37, 568.94] | [454.59, 589.40] | [447.71, 580.02] |
| Sex_B_ | [–131.41, 116.74] | [–111.25, 111.73] | [–113.42, 123.65] | [–105.39, 131.48] | [–110.55, 145.53] | [–151.13, 124.45] | [–129.70, 144.10] |
| SES_B_ | [21.10, 42.95] | [21.11, 42.11] | [23.24, 43.89] | [20.72, 42.80] | [22.55, 43.48] | [21.03, 42.18] | [20.85, 40.36] |
| IT_B_ |  | [–5.72, 8.69] | [–6.40, 8.09] | [–8.39, 5.16] | [–11.61, 2.70] | [–11.48, 2.23] | [–11.46, 1.83] |
| IT^2^_B_ |  | [–4.94, 4.04] | [–4.82, 4.33] | [–2.61, 8.49] | [0.05, 9.94] | [1.04, 9.88] | [0.43, 9.38] |
| IC_B_ |  |  | [–70.49, –0.25] | [–61.76, 26.74] | [–55.93, 30.45] | [–44.51, 86.59] | [–46.12, 84.84] |
| IT_B_$\times\mathrm{IC}$_B_ |  |  |  | [–14.82, 79.92] | [–14.81, 77.48] | [–17.66, 72.45] | [–31.44, 61.93] |
| IT^2^_B_$\times\mathrm{IC}$_B_ |  |  |  | [–76.73, 12.08] | [–92.95, -1.70] | [–93.80, –10.20] | [–93.29, –7.50] |
| HT2_B_ |  |  |  |  | [–7.71, 73.31] | [–15.04, 68.01] | [–13.71, 65.75] |
| HT3_B_ |  |  |  |  | [-146.43, 7.20] | [–141.59, 20.54] | [–132.48, 15.45] |
| HT4_B_ |  |  |  |  | [-173.79, 101.36] | [–189.11, 99.48] | [–176.43, 109.25] |
| HQ_B_ |  |  |  |  |  | [–84.43, 16.61] | [–121.75, 53.09] |
| HT2_B_$\times\mathrm{HQ}$_B_ |  |  |  |  |  |  | [–30.70, 478.77] |
| HT3_B_$\times\mathrm{HQ}$_B_ |  |  |  |  |  |  | [–278.68, 797.74] |
| HT4_B_$\times\mathrm{HQ}$_B_ |  |  |  |  |  |  | [–75.61, 69.53] |

# **Table S4**

**The 95% confidence intervals of standardized regression coefficients for the multilevel regression models predicting mathematics achievement**

| Variables | Model A0 | Model A1 | Model A2 | Model A3 | Model A4 | Model A5 | Model A6 |
| --- | --- | --- | --- | --- | --- | --- | --- |
| **Within-school level** |  |  |  |  |  |  |  |
| SEX_W_ | [–0.13, –0.06] | [–0.13, –0.06] | [–0.13, –0.06] | [–0.13, –0.06] | [–0.13, –0.06] | [–0.13, –0.06] | [–0.13, –0.06] |
| SES_W_ | [0.15, 0.25] | [0.15, 0.24] | [0.13, 0.22] | [0.13, 0.22] | [0.13, 0.22] | [0.13, 0.22] | [0.13, 0.22] |
| IT_W_ |  | [0.04, 0.14] | [0.04, 0.15] | [0.04, 0.14] | [0.04, 0.14] | [0.04, 0.14] | [0.04, 0.14] |
| IT^2^_W_ |  | [–0.12, –0.01] | [–0.11, –0.02] | [–0.12, –0.01] | [–0.12, –0.01] | [–0.11, –0.01] | [–0.12, –0.01] |
| IC_W_ |  |  | [0.14, 0.21] | [0.14, 0.22] | [0.15, 0.22] | [0.14, 0.24] | [0.14, 0.23] |
| IT_W_$\times\mathrm{IC}$_W_ |  |  |  | [–0.05, 0.04] | [–0.05, 0.04] | [–0.05, 0.04] | [–0.04, 0.05] |
| IT^2^_W_$\times\mathrm{IC}$_W_ |  |  |  | [–0.06, 0.02] | [–0.06, 0.02] | [–0.06, 0.02] | [–0.05, 0.03] |
| HT2_W_ |  |  |  |  | [0.02, 0.09] | [0.02, 0.09] | [0.02, 0.09] |
| HT3_W_ |  |  |  |  | [–0.03, 0.06] | [–0.03, 0.06] | [–0.04, 0.05] |
| HT4_W_ |  |  |  |  | [–0.03, 0.05] | [–0.03, 0.05] | [–0.03, 0.05] |
| HQ_W_ |  |  |  |  |  | [–0.05, 0.04] | [–0.03, 0.09] |
| HT2_W_$\times\mathrm{HQ}$_W_ |  |  |  |  |  |  | [–0.08, 0.02] |
| HT3_W_$\times\mathrm{HQ}$_W_ |  |  |  |  |  |  | [–0.08, –0.01] |
| HT4_W_$\times\mathrm{HQ}$_W_ |  |  |  |  |  |  | [–0.08, 0.01] |
| **Between-school level** |  |  |  |  |  |  |  |
| Intercept_B_ | [13.84, 28.97] | [14.43, 29.51] | [13.86, 27.54] | [13.88, 27.24] | [13.87, 26.74] | [14.50, 27.51] | [14.34, 26.60] |
| Sex_B_ | [–0.23, 0.21] | [–0.20, 0.20] | [–0.19, 0.21] | [–0.18, 0.22] | [–0.19, 0.25] | [–0.26, 0.21] | [–0.22, 0.25] |
| SES_B_ | [0.66, 0.97] | [0.67, 0.98] | [0.68, 0.98] | [0.61, 0.96] | [0.66, 0.99] | [0.61, 0.97] | [0.58, 0.93] |
| IT_B_ |  | [–0.21, 0.32] | [–0.23, 0.29] | [–0.30, 0.19] | [–0.43, 0.11] | [–0.42, 0.09] | [–0.42, 0.07] |
| IT^2^_B_ |  | [–0.25, 0.20] | [–0.23, 0.21] | [–0.14, 0.42] | [–0.01, 0.50] | [0.03, 0.50] | [0.00, 0.47] |
| IC_B_ |  |  | [–0.38, –0.00] | [–0.33, 0.14] | [–0.31, 0.17] | [–0.25, 0.48] | [–0.25, 0.46] |
| IT_B_$\times\mathrm{IC}$_B_ |  |  |  | [–0.08, 0.41] | [–0.08, 0.40] | [–0.10, 0.37] | [–0.16, 0.32] |
| IT^2^_B_$\times\mathrm{IC}$_B_ |  |  |  | [–0.60, 0.11] | [–0.73, 0.01] | [–0.75, –0.05] | [–0.77, –0.03] |
| HT2_B_ |  |  |  |  | [–0.04, 0.36] | [–0.07, 0.34] | [–0.07, 0.33] |
| HT3_B_ |  |  |  |  | [–0.29, 0.02] | [–0.28, 0.04] | [–0.26, 0.03] |
| HT4_B_ |  |  |  |  | [–0.27, 0.16] | [–0.29, 0.15] | [–0.27, 0.17] |
| HQ_B_ |  |  |  |  |  | [–0.54, 0.10] | [–0.78, 0.34] |
| HT2_B_$\times\mathrm{HQ}$_B_ |  |  |  |  |  |  | [0.03, 0.91] |
| HT3_B_$\times\mathrm{HQ}$_B_ |  |  |  |  |  |  | [–0.12, 0.35] |
| HT4_B_$\times\mathrm{HQ}$_B_ |  |  |  |  |  |  | [–0.03, 0.03] |

# **Table S5**

**Unstandardized coefficients and standard errors for the OLS regression models predicting mathematics interest**

| Variables | Model B0 | Model B1 | Model B2 | Model B3 | Model B4 | Model B5 | Model B6 |
| --- | --- | --- | --- | --- | --- | --- | --- |
| **Within-school level** |  |  |  |  |  |  |  |
| SEX ($B_{01}$) | –0.20 (0.03) ^***^ | –0.20 (0.03) ^***^ | –0.21 (0.02) ^***^ | –0.21 (0.02) ^***^ | –0.21 (0.02) ^***^ | –0.19 (0.02) ^***^ | –0.19 (0.02) ^***^ |
| SES ($B_{02}$) | 0.08 (0.02) ^***^ | 0.08 (0.02) ^***^ | 0.02 (0.02) | 0.02 (0.02) | 0.02 (0.02) | 0.02 (0.02) | 0.02 (0.02) |
| IT ($B_{10}$) |  | –0.03 (0.01) ^*^ | –0.02 (0.01) | –0.02 (0.01) | –0.02 (0.01) | –0.01 (0.01) | –0.01 (0.01) |
| IT^2^ ($B_{20}$) |  | 0.00 (0.00) | 0.00 (0.00) | 0.00 (0.00) | 0.00 (0.00) | 0.00 (0.00) | 0.00 (0.00) |
| IC ($B_{03}$) |  |  | 0.79 (0.03) ^***^ | 0.79 (0.03) ^***^ | 0.79 (0.03) ^***^ | 0.45 (0.04) ^***^ | 0.45 (0.04) ^***^ |
| IT$\times\mathrm{IC}$ ($B_{13}$) |  |  |  | 0.05 (0.02) ^*^ | 0.05 (0.02) ^*^ | 0.04 (0.02) ^*^ | 0.05 (0.02) ^*^ |
| IT^2^$\times\mathrm{IC}$ ($B_{23}$) |  |  |  | –0.00 (0.01) | –0.00 (0.01) | –0.01 (0.01) | –0.01 (0.01) |
| HT2 ($B_{30}$) |  |  |  |  | –0.03 (0.03) | –0.01 (0.03) | –0.01 (0.03) |
| HT3 ($B_{40}$) |  |  |  |  | –0.12 (0.06) | –0.09 (0.06) | –0.10 (0.06) |
| HT4 ($B_{50}$) |  |  |  |  | –0.23 (0.10) ^*^ | –0.17 (0.09) | –0.17 (0.09) |
| HQ ($B_{04}$) |  |  |  |  |  | 0.48 (0.03) ^***^ | 0.51 (0.04) ^***^ |
| HT2$\times\mathrm{HQ}$ ($B_{34}$) |  |  |  |  |  |  | –0.08 (0.04) |
| HT3$\times\mathrm{HQ}$ ($B_{44}$) |  |  |  |  |  |  | –0.13 (0.09) |
| HT4$\times\mathrm{HQ}$ ($B_{54}$) |  |  |  |  |  |  | –0.01 (0.09) |
| **Between-school level** |  |  |  |  |  |  |  |
| Intercept_B_ ($\gamma_{00}$) | 3.17 (0.03) ^***^ | 3.17 (0.03) ^***^ | 3.20 (0.02) ^***^ | 3.20 (0.02) ^***^ | 3.22 (0.02) ^***^ | 3.21 (0.02) ^***^ | 3.21 (0.02) ^***^ |

*Note.* ^*^*p* < 0.05, ^**^*p* < 0.01, ^***^*p* < 0.001. The values presented in the tables are unstandardized regression coefficients, with standard errors in parentheses. HT1–HT4 are dummy-coded variables for homework time categories. HT1 was used as the reference category.

# **Table S6**

**The 95% confidence intervals of unstandardized regression coefficients for the OLS regression models predicting mathematics interest**

| Variables | Model A0 | Model A1 | Model A2 | Model A3 | Model A4 | Model A5 | Model A6 |
| --- | --- | --- | --- | --- | --- | --- | --- |
| **Within-school level** |  |  |  |  |  |  |  |
| Intercept | [4.14, 4.46] | [3.13, 3.25] | [3.17, 3.27] | [3.17, 3.27] | [3.18, 3.29] | [3.17, 3.27] | [3.17, 3.27] |
| SEX | [–0.26, –0.15] | [–0.26, –0.15] | [–0.26, –0.17] | [–0.26, –0.17] | [–0.26, –0.17] | [–0.23, –0.15] | [–0.23, –0.15] |
| SES | [0.03, 0.12] | [0.04, 0.12] | [–0.01, 0.06] | [–0.01, 0.06] | [–0.01, 0.06] | [–0.01, 0.05] | [–0.01, 0.05] |
| IT |  | [–0.06, –0.01] | [–0.05, 0.00] | [–0.05, 0.00] | [–0.04, 0.01] | [–0.04, 0.01] | [–0.04, 0.01] |
| IT^2^ |  | [–0.01, 0.01] | [–0.00, 0.01] | [–0.00, 0.01] | [–0.00, 0.01] | [–0.00, 0.01] | [–0.00, 0.01] |
| IC |  |  | [0.73, 0.85] | [0.73, 0.86] | [0.72, 0.85] | [0.37, 0.53] | [0.37, 0.53] |
| IT$\cdot IC$ |  |  |  | [0.01, 0.09] | [0.00, 0.09] | [0.01, 0.08] | [0.01, 0.08] |
| IT^2^$\cdot\mathrm{IC}$ |  |  |  | [–0.02, 0.01] | [–0.02, 0.01] | [–0.02, 0.01] | [–0.02, 0.01] |
| HT2 |  |  |  |  | [–0.08, 0.03] | [–0.07, 0.04] | [–0.07, 0.04] |
| HT3 |  |  |  |  | [–0.24, 0.00] | [–0.21, 0.03] | [–0.22, 0.02] |
| HT4 |  |  |  |  | [–0.42, –0.03] | [–0.35, 0.01] | [–0.35, 0.02] |
| HQ |  |  |  |  |  | [0.41, 0.54] | [0.43, 0.59] |
| HT2$\cdot HQ$ |  |  |  |  |  |  | [–0.16, 0.00] |
| HT3$\cdot\mathrm{HQ}$ |  |  |  |  |  |  | [–0.30, 0.04] |
| HT4$\cdot\mathrm{HQ}$ |  |  |  |  |  |  | [–0.19, 0.17] |
| **Between-school level** |  |  |  |  |  |  |  |
| Intercept | [3.12, 3.22] | [3.12, 3.21] | [3.17, 3.24] | [3.17, 3.24] | [3.19, 3.26] | [3.17, 3.24] | [3.17, 3.24] |

# **Table S7**

**The 95% confidence intervals of standardized regression coefficients for the OLS regression models predicting mathematics interest**

| Variables | Model A0 | Model A1 | Model A2 | Model A3 | Model A4 | Model A5 | Model A6 |
| --- | --- | --- | --- | --- | --- | --- | --- |
| **Within-school level** |  |  |  |  |  |  |  |
| SEX ($B_{01}$) | [0.04, 0.15] | [0.04, 0.15] | [–0.01, 0.07] | [–0.01, 0.07] | [–0.01, 0.07] | [–0.01, 0.06] | [–0.01, 0.06] |
| SES ($B_{02}$) |  | [–0.12, –0.01] | [–0.09, 0.01] | [–0.09, 0.01] | [–0.09, 0.01] | [–0.07, 0.02] | [–0.07, 0.01] |
| IT ($B_{10}$) |  | [–0.03, 0.06] | [–0.02, 0.05] | [–0.01, 0.06] | [–0.01, 0.06] | [–0.01, 0.05] | [–0.01, 0.05] |
| IT^2^ ($B_{20}$) |  |  | [0.51, 0.59] | [0.51, 0.60] | [0.51, 0.60] | [0.26, 0.38] | [0.26, 0.38] |
| IC ($B_{03}$) |  |  |  | [0.01, 0.10] | [0.00, 0.10] | [0.01, 0.08] | [0.01, 0.09] |
| IT$\cdot IC$ ($B_{13}$) |  |  |  | [–0.06, 0.03] | [–0.06, 0.03] | [–0.06, 0.03] | [–0.06, 0.02] |
| IT^2^$\cdot\mathrm{IC}$ ($B_{23}$) |  |  |  |  | [–0.05, 0.02] | [–0.04, 0.02] | [–0.04, 0.02] |
| HT2 ($B_{30}$) |  |  |  |  | [–0.08, 0.00] | [–0.07, 0.01] | [–0.07, 0.01] |
| HT3 ($B_{40}$) |  |  |  |  | [–0.09, –0.01] | [–0.08, 0.00] | [–0.08, 0.00] |
| HT4 ($B_{50}$) |  |  |  |  |  | [0.33, 0.43] | [0.35, 0.47] |
| HQ ($B_{04}$) |  |  |  |  |  |  | [–0.07, 0.00] |
| HT2$\cdot HQ$ ($B_{34}$) |  |  |  |  |  |  | [–0.07, 0.01] |
| HT3$\cdot\mathrm{HQ}$ ($B_{44}$) |  |  |  |  |  |  | [–0.04, 0.03] |
| HT4$\cdot\mathrm{HQ}$ ($B_{54}$) |  |  |  |  |  |  |  |
| **Between-school level** | [14.08, 24.92] | [14.84, 25.56] | [20.46, 50.86] | [20.66, 50.02] | [21.68, 52.32] | [24.91, 64.58] | [24.60, 65.73] |
| Intercept | [0.04, 0.15] | [0.04, 0.15] | [–0.01, 0.07] | [–0.01, 0.07] | [–0.01, 0.07] | [–0.01, 0.06] | [–0.01, 0.06] |


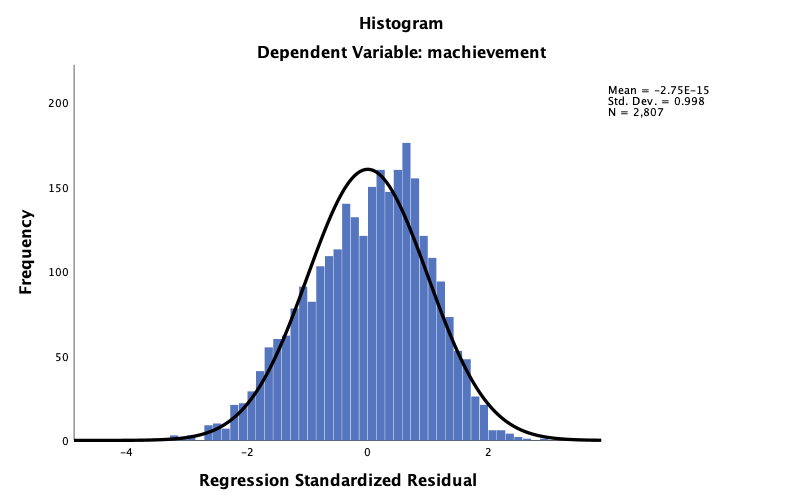


# **Figure S1**

**The histograms of standardized residuals for mathematics achievement**


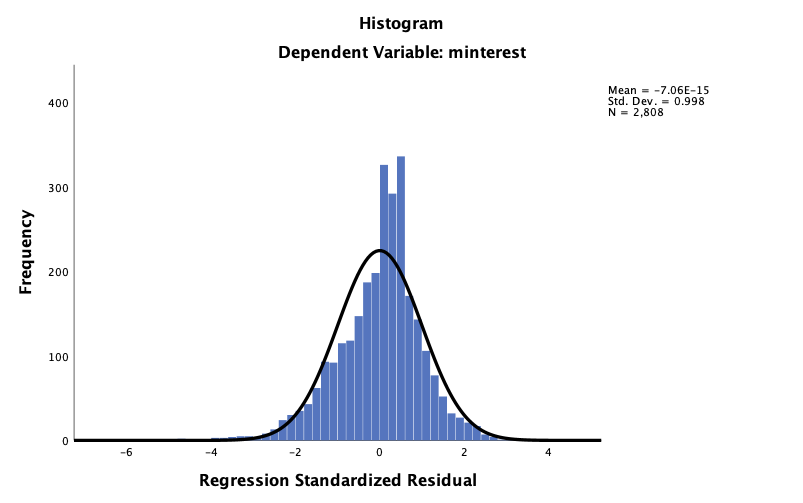


# **Figure S2**

**The histograms of standardized residuals for mathematics interest**
